# Cell Death in Human Health and Disease

**DOI:** 10.1155/2014/243017

**Published:** 2014-07-16

**Authors:** Jianzhen Xu, Dong Wang, Wencai Ma

**Affiliations:** ^1^College of Bioengineering, Henan University of Technology, Zhengzhou 453000, China; ^2^College of Bioinformatics Science and Technology, Harbin Medical University, Harbin 150081, China; ^3^Department of Lymphoma and Myeloma, UT MD Anderson Cancer Center, Houston, TX 77054, USA

## 1. Introduction

Mammalian cells adopt several cellular mechanisms to die, including apoptosis, autophagy, and programmed necrosis. Cell death has an important homeostatic role, mediating the removal of damaged cells, organelles, and proteins. Accumulated studies have increasingly shown that cell death plays critical roles in a variety of human diseases including cancers, infection, and neurodegenerative diseases. Novel cell death based drug targets and predictive biomarkers have been evaluated in several clinical trials. This special issue summarizes some of the most recent advances in our understanding of the relationships between cell death pathways and human diseases.

## 2. Understanding the Molecular Mechanisms of Cell Death in Human Diseases

TNF-*α* induced neuroinflammation and neurotoxicity have been implicated in neurodegenerative diseases. However, the molecular mechanisms that lead to cytokine initiated neurotoxicity are not fully understood. Based on RIP3-deficient mice, S. Liu et al. reported that RIP3-mediated necroptosis is activated in the mouse hippocampus after intracerebroventricular injection of TNF-*α*. They demonstrated that the signaling pathway CYLD-RIP1-RIP3-MLKL plays an important role in TNF-*α* induced necrosis, while neither ROS accumulation nor calcium influx is critical for the execution of cell death. Their work provided the first* in vivo* evidence for a role of RIP3 in TNF-*α* induced neurotoxicity in hippocampal neurons.

Dysregulation of cell death pathway is one of the hallmarks of cancers. In osteosarcoma U2OS cells, P. Chen et al. found that estrogen-related receptor alpha (ERR) blocked methotrexate-induced cell death and attenuated the activation of p53-mediated apoptosis signaling pathway. Their results provided a novel molecular understanding of methotrexate resistance and identified potential treatment strategies for osteosarcoma.

Resveratrol has been used as a supplemental treatment for several neurological and nonneurological diseases. But it is not clear whether resveratrol has neuroprotective effect on amyotrophic lateral sclerosis (ALS), a fatal neurodegenerative disease. L. Song et al. found that resveratrol treatment attenuated motor neuron loss, reduced muscle atrophy, and improved mitochondrial function of muscle fibers in the ALS mice. Their findings suggested that resveratrol has antioxidant and antiapoptotic effects against ALS.

## 3. Summarizing the Recent Advances of Cell Death in Human Disease

In order to provide our readers with more comprehensive and deeper understanding of cell death in human diseases, we deliberately selected several nice review papers on this topic. In recent years, the fundamental regulatory roles of miRNAs have been linked to diverse physiological processes and human diseases. Focused on lung cancer, N. Othman and N. Hasima conducted a comprehensive survey about miRNAs in the apoptotic process and in sensitivity/resistance to common cancer treatments.

Cell death plays a multitiered immunological role in infection, inflammation, and immunity. In their review paper, T. Nunes et al. described the recent findings on the associations of cell death processes with the development of inflammatory bowel diseases in humans. They pointed out the complex crosstalk between autophagy, apoptosis, and necroptosis, which suggests that deeper understanding among the different forms of cell death is warranted.

Ubiquitination is a posttranslational modification in which ubiquitin is attached to proteins to modulate the degradation of substrate proteins via the proteasome and lysosome. Deubiquitinases (DUBs) regulate a variety of cellular processes by reversing ubiquitination. S. Bhattacharya and M. Ghosh summarized recent findings on the involvement of DUBs in cell death associated pathways related to cancers. This review provided a novel viewpoint of the regulation of cell death signaling.

Finally, our group contributed a review paper on cell death related biomarkers. We surveyed the current and emerging biomarkers involved in apoptosis, autophagy, and programmed necrosis and discussed their relationship with human diseases. This survey can help researchers to focus on the translation of laboratory discovery into clinical practice.

## 4. Prediction and Analysis of Cell Death Related Proteins and Gene Networks

Computational approaches play an increasing role in elucidating the components and molecular mechanisms of human cell death networks. J-proteins are molecular chaperones and can be classified into 4 types, that is, Type I, Type II, Type III, and Type IV. Different types of J-proteins play distinct roles in influencing cancer properties and cell death. Thus, reliably annotating the types of J-proteins is essential for better understanding of their molecular functions. P. Feng et al. proposed a support vector machine model to predict the four functional types of J-proteins based on reduced amino acid alphabet compositions. For the convenience of the experimental scientists, an online prediction server was also developed.

The formation and death of macrophages/foam cells are one of the major factors that cause coronary heart disease (CHD). Based on the Edinburgh human metabolic network (EHMN), X. Jia et al. built an enzyme-reaction network and proposed a computational approach to select modules related to programmed cell death. By integrating the subcellular location information with enzyme-reaction network, they identified the EHMN-mitochondria (EHMN-M) module, which was confirmed to be related to programmed cell death, CHD pathogenesis, and lipid metabolism via literature surveying. This method comprehensively analyzed CHD from the point of programmed cell death in subnetworks.

